# Quantitative Mass Spectrometry for Bacterial Protein Toxins — A Sensitive, Specific, High-Throughput Tool for Detection and Diagnosis

**DOI:** 10.3390/molecules16032391

**Published:** 2011-03-14

**Authors:** Anne E. Boyer, Maribel Gallegos-Candela, Renato C. Lins, Zsuzsanna Kuklenyik, Adrian Woolfitt, Hercules Moura, Suzanne Kalb, Conrad P. Quinn, John R. Barr

**Affiliations:** 1 Centers for Disease Control and Prevention, 4770 Buford Hwy, NE, Atlanta, GA 30341, USA; E-Mail: aboyer@cdc.gov (A.E.B.); 2 Battelle Analytical Services, Atlanta, at the Centers for Disease Control and Prevention, 4770 Buford Hwy, NE, Atlanta, GA 30341, USA; 3 Centers for Disease Control and Prevention, 1600 Clifton Rd., Atlanta, GA 30333, USA

**Keywords:** *Bacillus anthracis*, anthrax lethal factor, anthrax edema factor, *Clostridium botulinum*, neurotoxins, mass spectrometry

## Abstract

Matrix-assisted laser-desorption time-of-flight (MALDI-TOF) mass spectrometry (MS) is a valuable high-throughput tool for peptide analysis. Liquid chromatography electrospray ionization (LC-ESI) tandem-MS provides sensitive and specific quantification of small molecules and peptides. The high analytic power of MS coupled with high-specificity substrates is ideally suited for detection and quantification of bacterial enzymatic activities. As specific examples of the MS applications in disease diagnosis and select agent detection, we describe recent advances in the analyses of two high profile protein toxin groups, the *Bacillus anthracis* toxins and the *Clostridium botulinum* neurotoxins. The two binary toxins produced by *B. anthracis* consist of protective antigen (PA) which combines with lethal factor (LF) and edema factor (EF), forming lethal toxin and edema toxin respectively. LF is a zinc-dependent endoprotease which hydrolyzes specific proteins involved in inflammation and immunity. EF is an adenylyl cyclase which converts ATP to cyclic-AMP. Toxin-specific enzyme activity for a strategically designed substrate, amplifies reaction products which are detected by MALDI-TOF-MS and LC-ESI-MS/MS. Pre-concentration/purification with toxin specific monoclonal antibodies provides additional specificity. These combined technologies have achieved high specificity, ultrasensitive detection and quantification of the anthrax toxins. We also describe potential applications to diseases of high public health impact, including *Clostridium difficile* glucosylating toxins and the *Bordetella pertussis* adenylyl cyclase.

## 1. Introduction

Bacterial protein toxins are among the most potent natural poisons known. Members of this group of bioactive polypeptides can cause irreversible changes to host cellular targets resulting in a wide variety of functional losses, ranging from the paralyses caused by clostridial neurotoxins (flaccid paralysis or tonic spasms), to the immune collapse, endothelial dysfunction, hemorrhage and death due to the anthrax toxins [1,2]. Quantitative detection of specific toxin activity in clinical samples yields insights into the kinetics of intoxication, stage of infection, and pathogenesis associated with the presence and quantity of certain toxins. Rapid high-throughput analysis also has the potential to provide measurements that quantify the efficacy of toxin-based therapeutics and support patient management decisions during treatment. Methods recently developed in our laboratory have targeted the seven *Clostridium botulinum* neurotoxin types A-G and subtypes [3,4,5], and the *Bacillus anthracis* binary toxins [2,6,7].

The seven structurally related *C. botulinum* neurotoxin proteins (BoNTs) intoxicate the peripheral nervous system where they inhibit calcium-dependent secretion of acetylcholine at the neuromuscular junction and cause flaccid paralysis. The BoNTs are released from *C. botulinum* as single chain polypeptides that are post-translationally modified by protease hydrolysis to form covalently linked di-chain polypeptides, each with three discrete structural and functional domains. The common architecture of the BoNTs comprises an enzymatically active light chain (LC, 50 kDa) linked via a disulfide bridge to a receptor binding and translocating heavy chain (HC, 100 kDa). The HC is further comprised of two regions; the amino terminal 50 kDa (H_N_) domain with a translocation function and a 50 kDa carboxyl terminal 50 kDa domain (H_C_). The LC domains contain a zinc-dependent endoprotease activity which target one or more of the three membrane proteins involved in pre-synaptic cell secretory vesicle docking and membrane fusion, thus preventing neurotransmitter release. BoNTs /A, /C and /E hydrolyze synaptosomal associated protein 25 (SNAP-25), BoNTs B, D, F and G hydrolyze isoforms of synaptobrevin, also known as vesicle associated membrane protein (VAMP-2). BoNT/C is unique within the group in that it also hydrolyzes a second substrate, syntaxin, in the vesicle docking complex [8].

The seven BoNT serotypes A-G have considerable sequence diversity as well as regions of homology between toxin types. In addition, several toxin subtypes have been described for each toxin type [9]. As described, the active BoNT consists of both the HC responsible for binding the cellular receptor and translocating the LC that carries the endoproteolytic activity [10]. At the extremes in diversity are the BoNT/C and BoNT/D toxins which have combined through evolution to form mosaic toxins that are mixtures of type /C and type /D neurotoxins [11]. Extensive characterization of these toxins has revealed that these mosaic toxins are either consistent with a type /C heavy chain and /D light chain, or a /D heavy chain and /C light chain [12]. Differentiation of the toxin type is clinically critical for ensuring treatment with the appropriate anti-toxin [5,13].

**Figure 1:**
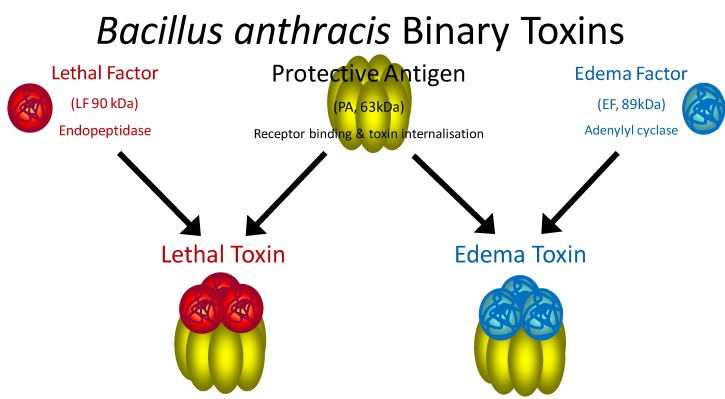
*Bacillus anthracis* secretes three toxin-associated proteins, lethal factor (LF), protective antigen (PA), and edema factor (EF) which form the two binary anthrax toxins. Protective antigen (PA_83_) is an 83 kDa protein responsible for toxin entry into the cell. PA binds receptors at the cell surface where it is cleaved by a furin-like protease removing a 20 kDa portion. The remaining 63 kDa PA_63_ forms an oligomer at the cell surface that binds three molecules of lethal factor (LF), a zinc-endopeptidase, and edema factor (EF), adenylylcyclase, forming lethal toxin and edema toxin respectively.

*Bacillus anthracis* is a Gram positive spore-forming rod. Exposure to the spores typically occurs via three routes: 1) dermal contact results in cutaneous anthrax, 2) ingestion results in gastrointestinal anthrax, and 3) inhalation results in the most deadly form, pulmonary anthrax. When spores enter the body, they germinate, enter the vegetative growth phase and begin to produce toxin. The anthrax toxins are secreted as three distinct proteins and their activities have been well described [14,15]. Protective antigen (PA) is secreted as an 83 kDa protein that binds to cell surface receptors and is cleaved by furin-like protease releasing a 20 kDa portion and retaining the 63 kDa form at the cell surface (PA_63_) [16,17]. PA_63_ forms an oligomer comprising up to 8 molecules of PA_63_ and is responsible for toxin internalization [18]. It combines with lethal factor (LF) and edema factor (EF), forming lethal toxin (LTx) and edema toxin (ETx) respectively [14] (Figure 1). LF is similar to the BoNT’s in that it is a zinc-dependent endoprotease. The substrate for LF hydrolysis however, is the mitogen activated protein kinase kinase (MAPKK) family of response regulators centrally involved in inflammation and immunity [15]. EF is a calmodulin-dependent adenylyl cyclase that converts ATP to cyclic AMP (cAMP). Elevated cAMP leads to edema and immune suppression. Both toxins synergize to cause immune dysregulation, endothelial dysfunction, advanced septicemia, hemorrhage, and shock which often leads to death [19,20].

Our work has exploited customized peptide substrates that enable accumulation of stable peptide hydrolysis products for MS detection. This accumulation serves to amplify the reporter signal capacity of the enzymatic activities of specific toxins. Combined with MS detection, the result is an analytically ultra-sensitive method (attomoles/mL) for detection and quantification of toxin activities in clinical samples. Toxin-activity targeted quantification has many benefits. The first is the capacity for amplification and increased sensitivity with specific toxin selection. Rather than detecting molecular levels of a substance, protein, or enzyme toxin in the blood, detecting the toxins activity in the presence of excess substrate allows accumulation of the enzyme-specific reaction products over time. Furthermore, detection of toxin-specific reaction products may often be easier and simpler than detection of the toxin itself. An additional benefit to the toxin-activity/analytical detection approach includes substrate selection. The substrate may be strategically designed to enhance both activity and analytical detection of the resulting reaction products. Also, selecting toxins over other molecular diagnostic targets may be important since toxin activities are often clinically and pathologically relevant. This was demonstrated for the anthrax toxin LF levels that were inversely associated with survival time during experimental inhalation anthrax [21]. In addition, the exotoxins are secreted factors that do not remain associated with the microorganisms. For example, targeting the toxin is essential for detecting botulism, because it is generally a consequence of ingesting or adsorbing pre-formed toxin and is not usually the result of an infection, except for infant and adult colonization botulism. For anthrax and other infections, toxin detection is important because of the potential for antimicrobial or immunological clearance of the organism, which was shown to occur during experimental inhalation anthrax [6] and in clinical inhalation anthrax [22]. In human inhalation anthrax, LF was not cleared and remained detectable in the blood for 12 days after antimicrobial treatment [22]. During experimental anthrax infection, diagnostic tests that depended on detection of the organism (culture) or its DNA (PCR) were negative within 24 hours after the initiation of antimicrobial therapy [21]. Additionally, secreted toxins are often produced at high levels during infection and therefore, may accumulate in blood or other clinical samples. LF was detected in the blood of rhesus macaques as early as 12 hours after inhalation exposure to *B. anthracis* Ames spores [21]. Depending on the route of infection or intoxication, toxins are often the most viable, relevant, and abundant target.

**Figure 2:**
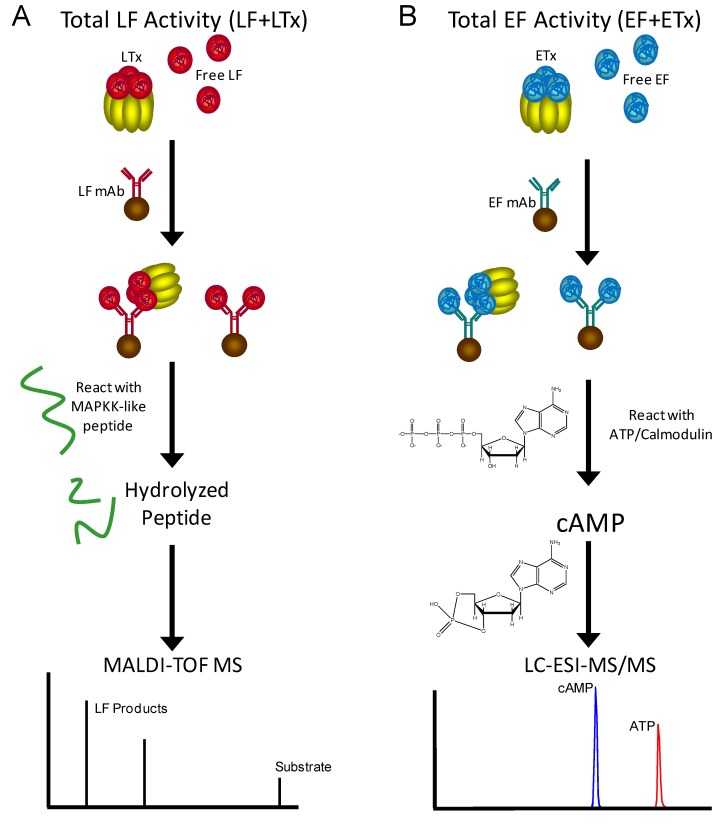
Schematic for total LF (LF+LTx) (A) and total EF (EF+ETx) detection by mass spectrometry. LF is captured by monoclonal antibodies (mAbs), mixed with a synthetic peptide substrate that is hydrolyzed, then the remaining substrate and two hydrolysis products are detected by MALDI-TOF MS. EF is captured by mAbs, then incubated with ATP and the adenylylcyclase cofactor calmodulin, then ATP and the adenylylcyclase reaction product cAMP are detected by LC-ESI-MS/MS.

Analytical MS-based detection is one of the important features of these toxin activity/reaction product targeted methods. MS is unique in that it provides additional assurance of specificity compared to ELISA colorimetric or fluorescent assays which sometimes exhibit cross reactivity [23]. Our methods for toxin activity quantification by MS incorporate three levels of specificity and sensitivity that provide unparalleled diagnostic assurance. The three levels include 1) toxin purification/ enrichment using monoclonal antibodies (mAbs) specific for the toxin that are covalently bound to magnetic beads, 2) toxin-specific enzyme activity directed against a specific substrate, and 3) mass specific detection of the toxin-generated reaction products. This method schematic is shown for MS detection of the anthrax toxins, LF and EF (Figure 2). LF and EF are found in the blood as both the monomers LF and EF and in complex with PA_63_ as LTx [24,25] and ETx, respectively. Therefore methods are developed using monoclonal antibodies that can capture both forms of LF and EF and include the total LF (LF + LTx) and total EF (EF + ETx). Briefly, the total LF is captured from a clinical sample using LF-specific monoclonal antibodies bound to magnetic beads (Figure 2A). The captured LF is then exposed to a MAPKK-like peptide substrate that it hydrolyzes at a specific residue yielding two products of a specific mass. The remaining substrate and two LF-specific products are detected by MALDI-TOF MS (Figure 2A). The same method flow has been followed to develop quantitative methods for EF adenylyl cyclase activity using the LC-ESI-MS/MS platform, which is preferred for detection of the small molecules represented by EF’s substrate ATP and catalytic product cAMP (Figure 2B).

## 2. Mass Spectrometric Detection of Toxin Activity

### 2.1. MALDI-TOF MS for peptides

Detection of the activity of endoproteolytic toxins such as the botulinum neurotoxins and LF is typically performed by MALDI-TOF MS because it is high-throughput (analyzes a 384-spot plate in less than 45 minutes), sensitive and robust. After the endoprotease peptide substrate cleavage reaction, no sample purification is needed. Samples are mixed with a chemical matrix consisting of α-cyano-4-hydroxycinnamic acid (CHCA) and spotted on the plate where it co-crystallizes with the sample. A 337 nm nitrogen laser strikes the chemical matrix containing sample on each spot. This ionizes and desorbs the peptides from the plate (laser desorption ionization) and a voltage potential is applied that propels the ions down a flight tube where they are separated by mass-to-charge (*m/z*) ratio (time-of-flight). Since all ions have the same energy, peptide ions with a smaller *m/z* ratio travel faster and are first to the detector, while ions with larger *m/z* ratio are slower (E = mv^2^). The instrument calibration accounts for these differences in *m/z* ratios with mass calibration standards. The spectra are high resolution (10,000–14,000) and the isotopic distributions associated with each peptide are distinct, easily visible, and can be observed in subsequent figures. MALDI-TOF MS is highly sensitive and often detects peptides less than 4,000 *m/z* in the femtomolar range. The high sensitivity of product detection is important because some toxins, such as BoNTs, are highly potent and very little is required to produce fully-paralytic botulism. MALDI-TOF MS also allows very early detection of the anthrax toxins, within 12–24 h after exposure to spores, when toxin levels are still quite low. In addition, the MALDI-TOF MS method for anthrax toxins are fast, providing results in less than 4 hours, and high-throughput, analysis of a 384-spot MALDI plate can be completed in less than 45 minutes. As observed in the 2001 anthrax letter attacks, the fatality rate was 45% despite the use of antibiotics and aggressive supportive care [22]. Early detection is essential for improving survival with conventional treatment. The sensitive, fast, MS-based toxin methods may provide the early diagnosis required for increasing survival rates.

**Figure 3:**
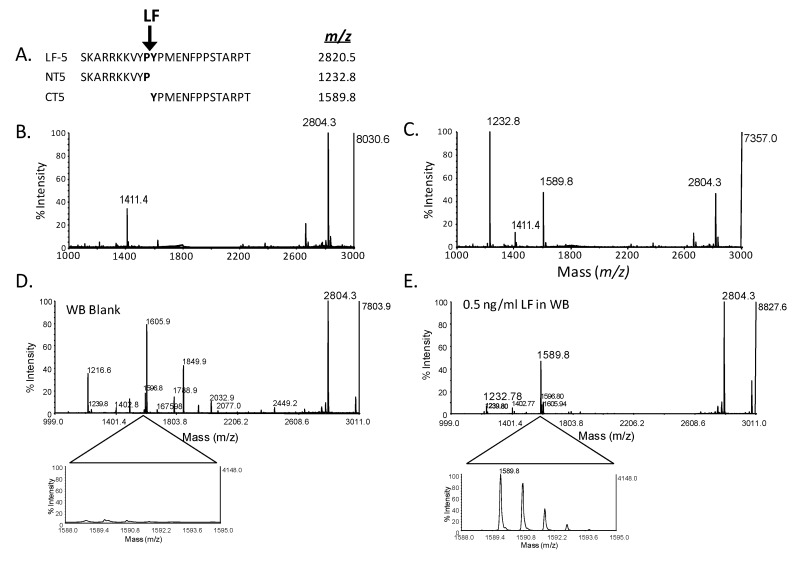
Specificity of MALDI-TOF MS detection of LF hydrolytic activity. Amino acid sequence and expected mass/charge (*m/z*) of lethal factor (LF) endoproteolytic MAPKK-like substrate (LF-5) and LF-specific hydrolysis products; amino-terminal product (NT5) and carboxy-terminal product (CT5) (A). MALDI-TOF MS of 2 nmoles LF-5 substrate and reaction buffer without LF (B) and with 1 ng LF (C). Both reactions, with and without LF, were incubated for 2 hours at 37 °C, and analyzed by MALDI-TOF MS scanning from 1,000–3,000 *m/z*. MALDI-TOF MS of 200 µL whole blood without LF (WB blank) (D) and spiked with 0.5 ng/mL LF (E) and purified with LF mAbs covalently bound to tosyl-activated magnetic beads, incubated overnight in a reaction buffer containing 2 nmoles substrate. Narrowing the *m/z* range on the CT5 (*m/z* 1,589.8) product shows the specificity for LF cleavage without LF (D) and with LF (E). Signal intensity for the CT5 focused mass window for WB blank was normalized to the focused spectrum for the 0.5 ng/mL LF spiked in WB.

A simple MALDI-TOF MS method for scanning masses in the appropriate mass range can visualize differences in toxin substrate reactivity, reaction mixture components, and antibody capture optimization. MALDI-TOF MS delivers a MS fingerprint of most peptides in a mixture. Thus, it can easily visualize substrate hydrolysis as well as the introduction of potential interferences during method development. This is shown for a simple reaction without and with LF added to reaction buffer with a synthetic peptide substrate, designed as an enhanced surrogate to its natural MAPKK substrate (Figure 3). The sequence of the peptide substrate (LF-5) has an expected mass of 2,804.2 *m/z*. Cleavage of LF-5 by LF would yield two smaller product peptides of expected masses: amino terminal product (NT5) of 1,231.8 *m/z* and carboxy terminal product (CT5) of 1,589.8 *m/z* (Figure 3A). MALDI-TOF MS for pure reactions (in the absence of a sample matrix such as serum and without antibody purification) was very clear. In the absence of LF, the full mass spectrum shows that only the singly and doubly charged substrate peaks are present (Figure 3B). In the presence of 1 ng LF, the substrate peak is reduced and two LF-specific product peaks of the expected masses are present (Figure 3C).

In addition to assessing adequate substrate hydrolysis under perfect conditions without sample interferences, the mass specificity and total fingerprint view of the catalytic reaction mixture allows MALDI-TOF MS to assess potential cross reactivity. For clinical samples, the abundance of proteases in serum and other clinical matrices may cause non-specific substrate degradation, yielding many smaller peptide products of different masses. MALDI-TOF MS readily detects this type of non-specific peptide hydrolysis. Figure 3D depicts a MALDI-TOF MS of a whole blood (WB) negative control (without LF) after purification by LF mAbs on magnetic beads and incubation with LF5 by methods described previously [6,7]. The spectrum shows many peaks associated with non-specific protease activity (Figure 3D). However, in WB containing 0.1 ng LF, cleavage of LF5 yields the LF-specific product peaks at 1,232.8 and 1,589.8 *m/z*, in addition to the non-specific peaks which are reduced due to abundant LF-specific cleavage (Figure 3E). Narrowing the *m/z* range allows visualization of the CT5-associated peak which is minimal in WB without LF and has high peak intensity in WB spiked with LF (Figure 3D, E). Our combination of procedures for detection of LF and BoNT activity are optimized to eliminate residual protease activity and are described below. Figure 3 shows that even under the most extreme conditions, MS provides reliable specificity and detects the difference between samples containing LF and those without LF, effectively ruling out non-specific cleavage.

### 2.2. Isotope dilution quantification for MALDI-TOF MS

Isotope dilution refers to the inclusion of an isotopically labeled, synthesized version of the target analyte with the sample for analysis. It serves as an internal standard (IS) to account for potential error related to sample processing and analysis. Isotope dilution improves the accuracy and precision of MS based quantification and is particularly important for MALDI-TOF MS quantification methods because of the co-crystallization process, for which stable isotope labeled internal standards co-crystallize with the native analytes. It has previously been described in detail for tandem MS (MS/MS) quantification [26,27]. In the case of LF activity, for the substrate LF4, the two internal standards are synthetic peptides, NT4-IS and CT4-IS, which each contain an isotopically labeled alanine (+7 mass units; with ^13^C_3_, ^15^N, D_3_) incorporated in place of the unlabeled alanine (Table 1). The IS sequences match those of the LF cleavage products, NT4 and CT4 (Table 1).

**Table 1:** The eight known LF protein targets aligned by their previously deduced cleavage sites.The floating consensus shown is based on the one or two most abundant amino acids at a position. LF hydrolyzes these sequences between the P1 and P1’ residues (in bold print). MAPKK sequences for M1 (P29678), M2 (P36506), M3 (P46734), M4a and M4b (P47809), M6 (P52564), and M7a and M7b (O14733) from the National Center for Biotechnology Information (www.ncbi.nlm.nih.gov). LF-1 is a synthetic peptide based on the FRET-based substrate with enhanced cleavage compared to MAPKK-1 identified by Turk *et al* [30]. The core of LF-1 in parentheses is used for designing longer substrates, LF-2 to LF-4 and LF-S. Substrate and internal standard (IS) peptide sequences and [M + H]^+^ masses are listed.

Name					Sequence		[M + H]^+^																																							
P1 ↓ P1’
MAPKK-1_1-25_		(M1)			M	P	K	K	K	P	T	**P**		**I**	Q	L	N	P	A	P	D	G	S	A	V	N	G	T	S	S									
MAPKK-2_1-27_		(M2)	M	L	A	R	R	K	P	V	L	**P**		**A**	L	T	I	N	P	T	I	A	E	G	P	S	P	T	S	E									
MAPKK-3b_17-43_		(M3)	G	K	S	K	R	K	K	D	L	**R**		**I**	S	C	MS	K	P	P	A	P	N	P	T	P	P	R	N										
MAPKK-4_36-62_		(M4a)	S	M	Q	G	K	R	K	A	L	**K**		**L**	N	F	A	N	P	P	F	K	S	T	A	R	F	T	L	N									
MAPKK-4_49-75_		(M4b)	A	N	P	P	F	K	S	T	A	**R**		**F**	T	L	N	P	N	P	T	G	V	Q	N	P	H	I	E	R									
MAPKK-6_5-31_		(M6)	K	G	K	K	R	N	P	G	L	**K**		**I**	P	K	E	A	F	E	Q	P	Q	T	S	S	T	P	P	R									
MAPKK-7_35-61_		(M7a)	S	P	Q	R	P	R	P	T	L	**Q**		**L P**	L	A	N	D	G	G	S	R	S	P	S	S	E	S	S										
MAPKK-7_67-93_		(M7b)	P	P	A	R	P	R	H	M	L	**G**		**L P**	S	T	L	F	T	P	R	S M E S	I	E	I	D													
Consensus			S	P	A	R	R	K	K	T	L	**^P^/_K_**		**^L^/_I_ P**	L	^N^/_A_^N^/_P_^P^/_F_	^P^/_T_	P	A	S	T	P	S	P	T	S													
LF-1 (Core )						(R	R	K	K	V	Y	**P**		**Y**	P M	E) P	T	I	A																			1751.9	
LF-2				A	R	R	R	K	K	V	Y	**P**		**Y**	P M	E	P	T	I	A	K																	1880.1	
LF-3			S	P	A	R	R	K	K	V	Y	**P**		**Y**	P M	E	N	P	T	P	R	S	T	P	S	P	T											2857.5	
LF-4			S	K	A	R	R	K	K	V	Y	**P**		**Y**	P M	E	N	F	P	P	S	T	A	R	P	T												2821.5	
LF-5			S	K	A	R	R	K	K	V	Y	**P**		**Y**	P X	E	N	F	P	P	S	T	A	R	P	T												2804.2	
NT4/5			S	K			R	K	K	V	Y	**P**																										1232.8	
CT4						S								**Y**	P M	E	N	F	P	P	S	T	A	R	P	T												1607.8	
NT4/5-IS				K	(A+7)	R	R	K	K	V	Y	**P**																										1239.8	
CT4-IS														**Y**	P M	E	N	F	P	P	S	T	(A+7)	R	P	T												1614.8	
CT5														**Y**	P X	E	N	F	P	P	S	T	A	R	P	T												1589.8	
CT5-IS														** Y**	P X	E	N	F	P	P	S	T	(A+7)	R	P	T												1596.8	

**Figure 4:**
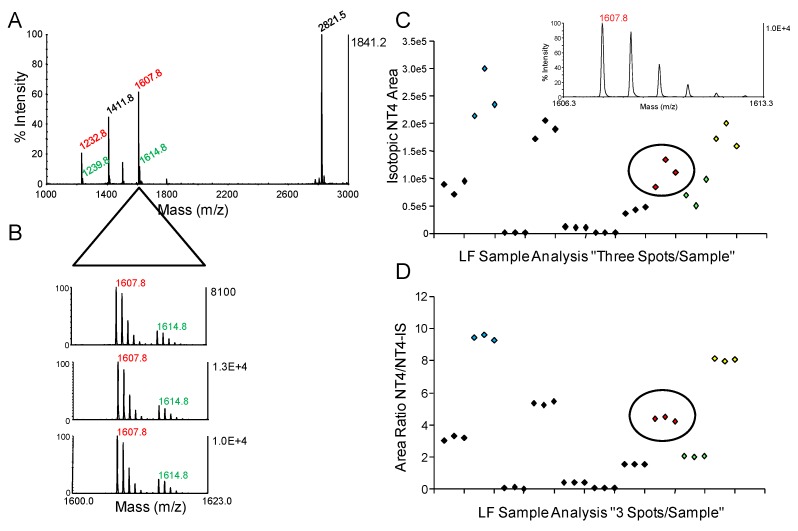
Isotope dilution for MALDI-TOF MS. Isotopically labeled internal standard peptides NT4-IS and CT4-IS, 7 mass units higher than the NT4 and CT4 peptide products are added to the sample and mixed in the MALDI chemical matrix CHCA as described previously [7]. A full spectrum is shown for a sample from an experimental rhesus macaque infection (A). The *m/z* range is narrowed for spectra from three spots of the same sample showing the CT4 LF cleaved product and the CT4-IS isotopic peaks and shows the variation in peak intensity from 8,092, 10,000 and 13,000 for the three spots (B). The CT4 peak areas alone were plotted for 10 archived samples from rhesus macaque anthrax inhalation infections and show the variation in area for triplet peaks from the same sample (C). The area ratios, area of CT4 divided by the area of CT4-IS, were plotted for the same 10 samples in C and shows how the variation was normalized (D). The areas and area ratios of the three spots in B were circled in C and D, respectively.

For MALDI-TOF MS quantification, each sample reaction mixture with IS is spotted on the MALDI plate with a minimum of three replicates and analyzed. With MALDI-TOF MS, considerable signal-to-signal variation in intensities occurs from spot-to-spot for the same sample mixture. Isotope dilution quantification is effective at normalizing these differences in spectrum intensities. Figure 4A shows the full MALDI-TOF mass spectrum for a cleaved LF substrate NT-4 (Table 1) produced from LF in an archived serum sample from rhesus macaques with inhalation anthrax infection [7]. Narrowing the mass range allows visualization of the target CT4 cleavage product and corresponding C-terminal internal standard (CT4-IS) (7 mass units higher than CT4) for three spectra acquired from individual spots from the same sample (Figure 4B). The intensity of the target analyte at 1,607.8 varies from 8,093 to 13,000. However, the ratios of the areas of the CT4/CT4-IS are similar. These differences were confirmed quantitatively for multiple samples containing different amounts of LF. Wide variation in the isotopic clustal areas of the CTP was observed for three replicates from the same samples prior to isotope dilution (Figure 4C). However, variation was minimized for the same three spots using the area ratio of the CT4/CT4-IS for normalization (Figure 4D).

### 2.3. LC-ESI-MS/MS for peptides

LC-ESI tandem MS (MS/MS) is also an appropriate platform for sensitive peptide detection and quantification. It is available in many laboratories and can be used as an alternative to MALDI-TOF MS for most peptide-based methods. In many cases, it is just as sensitive as MALDI-TOF MS and also requires no additional sample purification. One drawback however, is that LC-MS/MS has lower sample throughput. Depending on the optimized LC run conditions a single sample is analyzed in 5–10 minutes rather than in seconds as with MALDI-TOF MS. With MALDI-TOF MS, 96 samples can be analyzed in quadruplicate in less than 45 minutes. For LC-ESI-MS/MS, at least one hour is required to analyze 10 samples.

We further verified the use of MALDI-TOF MS for isotope dilution quantification by validating the LF method on the LC-ESI-MS/MS platform for potential application to public health laboratories housing this equipment, and reported the cross validation between the use of MALDI-TOF MS and LC-ESI-MS/MS for LF activity quantification [28]. We found that detection limits, accuracy and precision were very similar between the two methods. Importantly, we found a very close agreement between quantification of LF in the serum of New Zealand White rabbits with inhalation anthrax. Concentrations measured by ESI-MS/MS correlated with those by MALDI-TOF MS with a Pearson correlation coefficient of p = 0.99 and 9% mean difference, verifying the accuracy and interchangeability of both MS platforms for LF activity [28].

**Figure 5:**
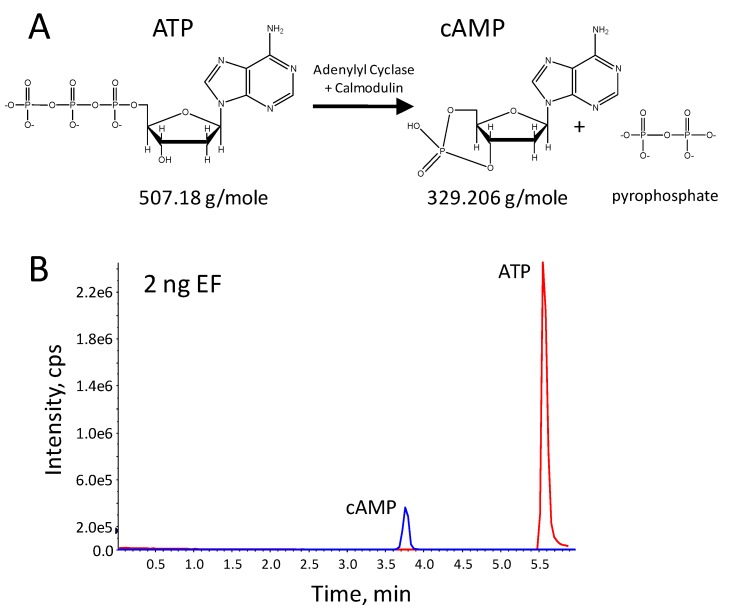
The structures of the edema factor (EF) substrate adenosine triphosphate (ATP) and reaction product cyclic adenosine monophosphate (cAMP) (A). Chromatogram for a 2 h reaction with 2 ng EF in the presence of 1 mM ATP generated by LC-ESI-MS/MS in positive ion mode, for the 508 ATP ion and 329/136 cAMP precursor/product (B).

### 2.4. LC-ESI-MS/MS for small molecules

MALDI-TOF MS is sometimes not appropriate for small molecule detection due to interferences from the chemical matrix at masses below 500 *m/z*. Therefore, LC-ESI-MS/MS is preferred for MS detection of the low mass substrate and products resulting from enzyme activities of the EF and the *Bordetella pertussis* adenylyl cyclase (PAC) toxins. In the presence of the cofactors calmodulin and calcium, EF and PAC convert adenosine triphosphate (ATP) to cyclic adenosine monophosphate (cAMP) (Figure 5A). The EF or PAC activity can be measured by the specific conversion of ATP to cAMP and loss of pyrophosphate. However, unlike the high substrate specificity of LF and the BoNTs, there are adenylyl cyclases active in mammalian cell processes. Therefore, specificity is obtained using a prior antibody purification step specific for EF or PAC (Figure 2B).

Optimized separation and detection of ATP, cAMP (Figure 5A) and internal standard 2-chloro-cAMP was achieved using a Biobasic AX weak anion exchange HPLC column with 90-20% acetonitrile and pH 6.5–10 eluent gradient and a 6 min run time as described previously [29]. Multiple reaction monitoring in positive ESI mode for cAMP included the 329 *m/z* precursor ion and its 136, 312 and 232 *m/z* products, for the 2-chloro-cAMP internal standard the 345 *m/z* precursor ion and its 146, 327 and 247 *m/z* products, and for ATP the 508 *m/*z precursor and its 136 *m/z* product was monitored. As optimized, cAMP eluted at 3.8 min and ATP at 5.6 min (Figure 5B).

## 3. Toxin-Specific Substrate Design

### 3.1. Peptide substrate design

For endoprotease toxins such as botulinum neurotoxin and lethal factor, considerable information regarding known protein substrates is available in the literature [30,31,32,33]. LF’s known substrates include six family members of the MAPKK’s, MAPKK-1, -2, -3, -4, -6, and -7, which it cleaves near the N-terminus [33]. LF was shown to hydrolyze MAPKK-4 and MAPKK-7 in two locations [33]. The N-terminal sequences of the known MAPKK substrates are shown in Table 1. LF cleaves between the P1 and P1’ amino acids indicated. Residues at these positions are not strictly conserved among the MAPKK LF substrates, however, they may have certain requirements. For example, the P1 amino acid is more flexible since hydrophobic (P), hydrophilic (R, Q), amphiphathic (K) and neutral (G) amino acids are represented among the cleavable MAPKK’s. The P1’ requirements are more stringent, with primarily hydrophobic amino acids (A, I, L, F) represented at this position. Work by Benjamin Turk determined LF cleavage efficiencies for short 9-11 amino acid FRET peptides based on the MAPKK consensus sequence [30]. Applying a degenerate peptide library based approach to the short MAPKK consensus, amino acid substitutions were identified near the cleavage site that enhanced the catalytic efficiency of LF for the short peptide [30]. In the ‘enhanced’ MAPKK sequence, LF cleavage occurred at the core of a double tyrosine (Y) proline (P) pair (YPYP), cleaving at a P1 proline and atypical P1’ tyrosine. This sequence identification provided a means to enhance the catalytic reaction and improved detection limits for our LF MS assay.

In addition to promoting catalytic efficiency in substrate design, certain conserved sequences may need to be maintained. For example, a basic stretch of 2-5 arginines (R) and lysines (K) occur at the N-terminus of all MAPKK’s that are LF targets (Table 1). The basic stretch is absent in MAPKK-5 which is not cleaved by LF (not shown). In addition, the sequences distal to the cleavage site in MAPKK’s contain 2-6 prolines and 2-6 serines and/or threonines (Table 1). Our LF substrate was designed with the YPYP core and maintained these MAPKK derived consensus sequences (LF-3 and LF-4).

We previously investigated the ability of LF to cleave a series of peptide substrates [7]. One was a short peptide based on Turk’s enhanced FRET substrate without the donor/quencher FRET pair (LF-1) (Table 1). However, one drawback to LF-1 is that it lacks an ionizable amino acid in the potential LF-cleaved C-terminal (CT) product. Therefore, only the N-terminal (NT) LF cleavage product would be ionized and visible by MALDI-TOF MS. To produce a comparable substrate with two visible cleavage products, the second substrate (LF-2) contained an additional lysine (K) on the C-terminus (Table 1). An additional two peptides, LF-3 and LF-4 contained extended sequences based on the consensus.

**Figure 6:**
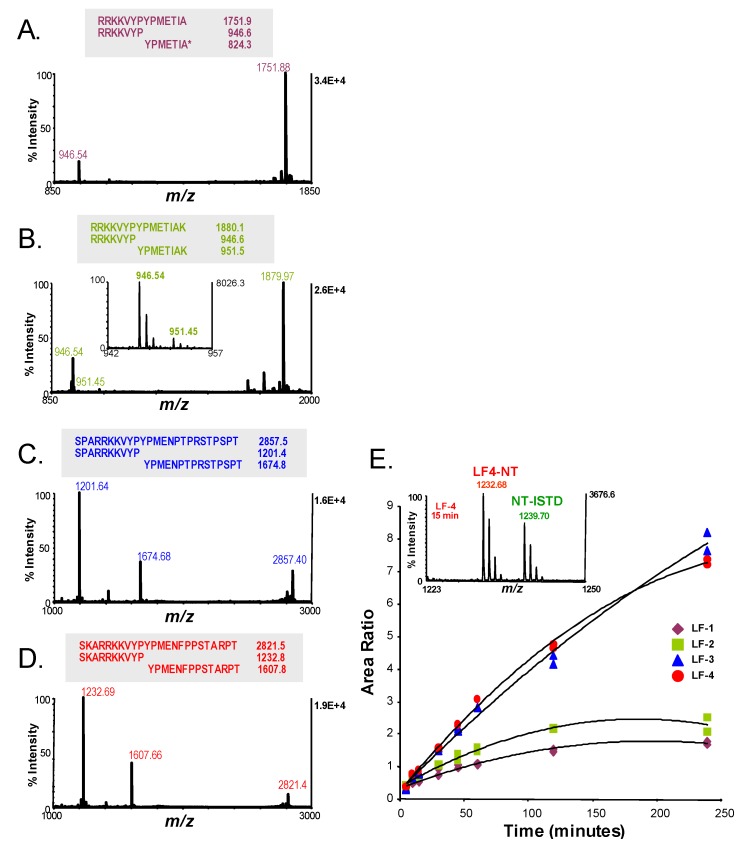
Comparison of cleavage efficiencies of lethal factor (LF) for various substrate peptides. Four peptides, LF-1, -2, -3, and -4, designed as potential substrates for LF, were compared to determine substrate qualities that produce enhanced LF hydrolysis. Full and expected LF cleaved peptide sequences and masses are shown for each substrate. Hydrolysis reactions for each substrate were conducted using 10 ng of LF, with 5 nmol of substrate in 200 µL reaction buffer, incubated for 2 h at 37 °C after which MALDI-TOF mass spectra were acquired without internal standard (A-D). The X-axis was narrowed to visualize both hydrolysis products for LF-2 which were separated by 5 mass units. The relative rates of LF hydrolysis of these substrates were compared for all 4 substrates (E) with timed reaction for each in 200 µL buffer and 10 ng LF, 5 nmol substrate, at 37 °C. MALDI-TOF mass spectra were acquired with internal standard (NT4-IS) at 5, 10, 15, 30, 45, 60, 90, 120, and 240 min. The area ratio of the NT to NT4-IS peptide peaks was plotted for each peptide and time point and fitted to a quadratic equation that depicted the relative rates of reaction for each substrate (E). The X-axis was narrowed for a representative spectrum from the LF hydrolysis of LF-4 at 15 min showing the NT-4 and NT4-IS peaks.

MALDI-TOF MS monitored the LF hydrolysis of all four peptides (LF-1 to LF-4) over time and their products were quantified as described previously (Figure 6) [7]. MALDI-TOF MS spectra after 2 hours reaction time with 10 ng LF are shown for each peptide substrate. As expected only the NT peak was visible for LF-1 at 946.5 *m/z*. Both NT and CT products at 946.5 and 951.5 *m/z* were visible in LF-2, due to the addition of the single lysine to the C-terminus of LF-1 (Figure 6B). This is clearly seen in the spectrum inset which focuses within a reduced mass window on both products. With the inset spectrum it can also be seen that the CT product of LF-2 has much lower intensity compared to the NT product due to the single ionizable amino acid in the CT product compared to four ionizable amino acids in the NT product (Figure 6B). LF-1 and LF-2 also have very low product intensities and very high substrate intensities (Figure 6A, B). In contrast, with the longer substrates, LF-3 and LF-4, the pattern is reversed with lower substrate intensities and higher product peak intensities, indicating greater substrate hydrolysis (Figure 6C, D).

The relative cleavage rates of these four substrates were determined by measuring the accumulation of hydrolysis products over time as previously described [7]. Briefly, the area ratios of the NT products for each peptide to an internal standard peptide (NT4-IS) were plotted *versus* reaction time (Figure 3E). NT4-IS is an isotopically labeled synthetic peptide that is 7 mass units higher than and with the same sequence as the LF-4 NT product, NT4 (Table 1). Accumulation of the NT hydrolysis products of the shorter peptides LF-1 and -2 was gradual out to 2 hours, after which no additional accumulation was observed (Figure 6E). In contrast, accumulation of NT products of LF-3 and LF-4 was faster and continued out to 4 hours. This substrate comparison suggests that the longer sequences were better substrates for LF since the hydrolysis products continued to accumulate over the 4 hour incubation. Greater product accumulation resulted in better sensitivity and detection limits.

In our current method we made an additional sequence substitution to improve the overall MS quantification. We substituted a norleucine (X) for the methionine in the CT product of LF-4 yielding peptide substrate LF-5 (Table 1). Methionines are easily oxidized; thus at very high levels of LF we observed a low-intensity peak representing the oxidized form of the LF-4 CT product, CT-4 (1,607.8 + 16 *m/z*). This 1,623.8 *m/z* peak represents the displacement of a small portion of CT4 from the quantitative 1,607.8 *m/z* peak. The simple substitution removed the opportunity for methionine oxidation. Thus, the total amount of product in the new substrate LF-5 was observed in the CT5 peak at 1,589.8 m/z, which is the main quantitative ion for the LF method. LF-5 had similar cleavage efficiency to LF-3 and -4 (data not shown). In all, the longer substrates were shown to be much better targets for LF hydrolysis. The same was observed for the botulinum neurotoxins and lengthened peptide substrates yielded higher amounts of cleavage products and lower detection limits (data not shown).

### 3.2. Substrate design for other toxin activities

Substrate design for other enzyme toxins depended on the catalytic properties. For example, substrate design and optimization was straightforward for the calmodulin-dependent adenylyl cyclase toxins including *B. anthracis* EF and the *B. pertussis* adenylyl cyclase (PAC) for which adenosine triphosphate (ATP) is considered the optimal substrate. It is likely that changes would reduce catalytic efficiency. For example, in our laboratory guanine triphosphate (GTP) was an inferior substrate (data not shown) and was demonstrated previously [34] indicating that a simple base change reduced the catalytic efficiency of EF.

*Clostridium difficile* has emerged as an important pathogen; it causes toxin-mediated illnesses ranging from mild diarrhea to fulminant pseudomembranous colitis in patients undergoing antimicrobial treatment and is the leading cause of hospital-associated diarrhea [35]. *C. difficile* toxins contributing to virulence include the glucosylating toxins, TcdA and TcdB, also known as Toxins A and B [36]. The toxin potency depends on their glucosyltransferase activity. Therefore, quantification of this activity in various strains associated with disease would be beneficial. The known substrates of TcdA and B include the small GTPases, Rho, Rac, and Cdc42, which are inactivated upon glucosylation of a threonine in position 37 in RhoA and 35 in Rac and Cdc42 [35]. It may be challenging to optimize substrates for MS detection of Tcd toxin activity as substrate specificity has only been shown for its full length GTPase polypeptides, which are not favorable for sensitive MS detection. We are investigating shorter peptides based on Rho, Rac and Cdc42 that include the Thr37/35 as possible substrates for glucosylation and MS detection.

## 4. Enzyme Reaction Optimization

### 4.1. Reaction buffer optimization of endoprotease/peptide substrate reactions

Optimization of conditions for the enzymatic reaction usually requires a standard buffer and mixture of metal ions. Hepes buffer at pH 7-8 was suitable for the enzyme reaction-based methods that were developed for LF, EF, and BoNTs [3,4,7,37]. However, for any enzyme, consideration of the reaction requirements including cofactors is essential. For the zinc-dependent endoproteases optimization of zinc (Zn^2+^) concentration is important and divalent ions, Ca^2+^, Mg^2+^ and/or Mn^2+^ may be important as well. The optimal reaction buffer composition for LF was 20 mM Hepes buffer pH 7.3., 1 mM DTT, 20 µM CaCl_2_, 10 mM MgCl_2_, 20 µM ZnCl_2_ [7]. In contrast, additional divalent metal cations CaCl_2_ and MgCl_2_ did not enhance BoNT activity, whereas bovine serum albumin (BSA) was important for catalytic efficiency of BoNTs in the absence of antibody capture [3].

For the LF and BoNT endoprotease peptide cleavage reactions, protease inhibitors were also required to maintain peptide substrate stability. This was especially important when analyzing clinical samples such as stool, for BoNT analysis, or whole blood for LF analysis. These samples contain abundant endogenous cysteine- and serine-proteases that can contribute to non-specific degradation of the substrate peptide (Figure 3A). Even with prior toxin purification some proteases may remain. Therefore, optimization and selection of protease inhibitors was critical. For evaluation, we selected protease inhibitors that did not inhibit the toxin activity. For metal ion-dependent hydrolytic enzymes, metal chelators such as ethylene diamine tetraacetic acid (EDTA) were not used, since even low concentrations exhibited a negative impact on EF and BoNT catalytic activity. Protease inhibitors and concentrations optimized for LF and BoNT reaction buffers included 0.56 mg/mL 4-(2-aminoethyl) benzenesulfonyl fluoride hydrochloride (AEBSF), 3.125 mg/mL 6-aminohexanoic acid, 0.3125 mg/mL antipain, 22 µg/mL (2*S*,3*S*)-*trans*-epoxysuccinyl-L-leucylamido-3-methylbutane ethyl ester (E-64D), and 0.125 mg/mL casein.

### 4.2. Reaction buffer optimization of adenylate cyclase activities

For the calcium and calmodulin dependent adenylyl cyclase family, which includes EF and PAC, reaction buffer optimization was completed using the JMP Statistical Discovery software ‘Design of Experiments’ protocol (JMP, Cary, NC) and yielded an optimal buffer formulation of 20 mM Hepes, pH 7.3, 0.1 mg/mL BSA, 1 mM EDTA, 10 mM CaCl_2_, 43 mM MgCl_2_, 1 µM calmodulin, and 0.05 mM ATP [29].

**Figure 7:**
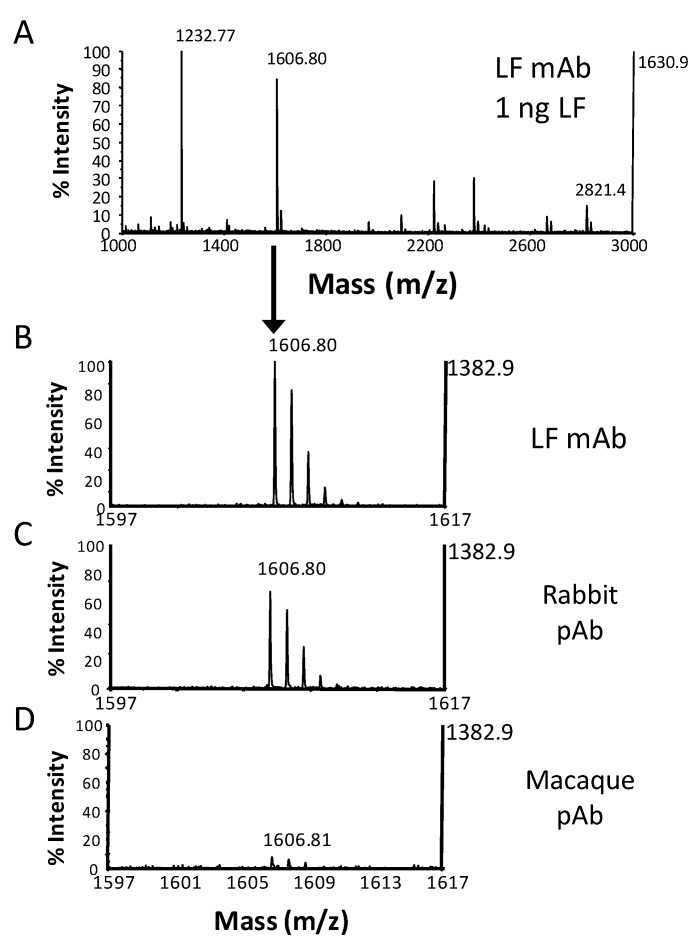
Mouse mAb’s, polyclonal serum (pAb) from rabbit immunized with LF, and pAb from rhesus macaque immunized with LF were covalently linked to protein G magnetic beads according to the manufacturer’s instructions (Invitrogen, Carlsbad, CA). All three antibodies on beads were used to capture 1 ng LF from serum, react with substrate LF-4 for 1.5 h, and MALDI-TOF MS was acquired according to procedures previously described [7]. The full mass spectrum is shown for the mouse LF mAb (A). MS focused on the CT4 LF cleavage product are shown for the mouse LF mAb (B), rabbit LF pAb (C), and macaque LF pAb (D).

## 5. Toxin Purification and Enrichment

The base of our toxin detection methods includes toxin concentration and purification using monoclonal antibodies (mAbs) crosslinked to Protein G or tosyl-activated magnetic beads. The magnetic bead extraction protocol resulted in significantly enhanced cleavage reactivity compared to crosslinking and capturing LF directly on a microtiter plate as is performed with typical enzyme-linked immunosorbent assays (ELISA). In the magnetic bead format there may be more steric freedom for interaction between antibody and toxin, and subsequent catalytic reactions. Magnetic beads, also provide an increased surface area for antibody loading on the bead, resulting in higher LF capture capacity.

Antibody selection for optimal toxin capture is another important criterion for method development. Although polyclonal antibodies (pAbs) are potentially useful, the myriad of possible epitopes they could recognize increases the probability that they will bind at or near the catalytic sight and interfere with the subsequent enzymatic reaction. For example, we compared the capture of 1 ng of LF using the LF mAb, polyclonal rabbit antisera to LF, and polyclonal rhesus macaque antisera to LF (Figure 7). Figure 7A shows the full MALDI-TOF MS from a 1.5 hour reaction of substrate LF-4 with LF captured by the LF mAb. In the spectrum focused on CT4, we observed a much greater accumulation of product from LF reactivity after capture by the LF mAb, a little less with the rabbit pAb, and very little product with the macaque pAb (Figure 7B-D). This indicates that the rhesus antisera had high toxin-neutralizing activity. For use in the final method, we selected two LF mAbs with no neutralizing activity, one of which was included in Figure 7A-B [38].

## 6. Toxin Quantification

**Figure 8:**
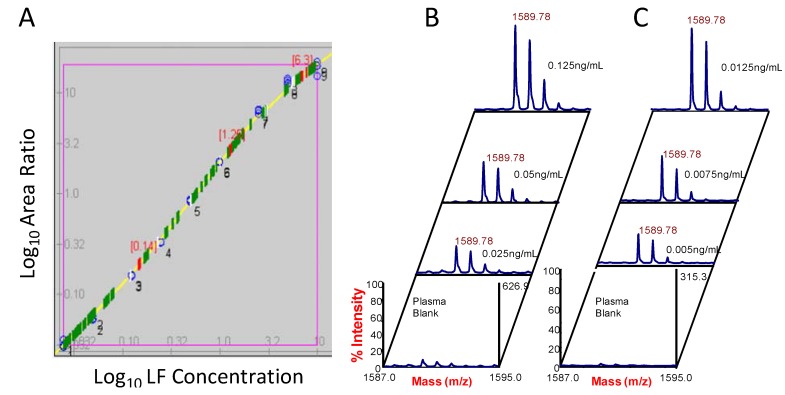
Standard curve for the low volume (20 µL) overnight incubation for standards ranging from 0.025 ng/mL to 10 ng/mL LF spiked in plasma, captured by LF mAbs on magnetic beads, and incubated with substrate LF-5, analyzed by MALDI-TOF MS, and CT-5 peaks quantified as described previously [6,7] (A). Spectra are shown with the X-axis narrowed on CT-5 LF hydrolysis product peak for high volume (200 µL) of LF standards; 0.125 ng/mL, 0.05 ng/mL, 0.025 ng/mL, and plasma blank control for 2 h reaction (B) and for 0.0125 ng/mL, 0.0075 ng/mL, and 0.005 ng/mL for the extended incubation (C).

The method development steps, toxin capture, catalytic reactivity, and mass spectrometry, combined, provide enhanced sensitivity and specificity compared to other diagnostic methods such as ELISA [39,40]. Incorporation of isotope dilution mass spectrometry also provides the ability to quantify toxin activity with a high degree of precision and accuracy. Coefficients of variation for LF quality control samples for 28 independent runs was 8.5–14.7% and accuracy for LF spiked samples ranged from 86 to 98.8% [38]. Serum or plasma standards were created by spiking recombinant LF at concentrations in the pre-optimized range. The spiked standards are subject to magnetic LF antibody bead capture, substrate cleavage reaction, and MALDI-TOF MS as described previously [6,7]. Concentrations optimized for LF using 20 µL of sample and 2 hour reaction incubation ranges from 0.125–25 ng/mL LF [38]. Extending the reaction overnight for the 20 µL sample volume yields an optimal quantifiable concentration curve range of 0.025–10 ng/mL (Figure 8A). Using a larger sample and standard volume, 200 µL, provides a similar concentration range and sensitivity of 0.025–10 ng/mL, in the shorter 2 h time period. Continuing the 200 µL reaction overnight yields an optimal range of 0.005–1 ng/mL. The lowest three standards and plasma blank are shown for the 200 µL 2 h reaction (Figure 8B) and continued overnight reaction (Figure 8C). These spectra show the rapid, less than 4 hour time to detection limit of 0.025 ng/mL and the detection limit for the extended reaction time of 0.005 ng/mL (Figure 8B,C).

Custom in-house designed Visual Basic programs allow conversion of the MALDI-TOF T2D-generated mass spectra to text files. These text files are then manipulated to sum the isotopes and produce a single chromatogram peak format for peak area determination. Area ratios of the target CT-5 analyte peak to CT5-IS are plotted *versus* the LF concentration on a log_10_-log_10_ scale. The standard curves generated are primarily linear in the central range and deviate slightly at the upper and lower bounds. Therefore, a 4-point sliding linear fit is incorporated which is stitched (Figure 8A) and algorithms are used to calculate unknown sample values. LF quantification has been validated independently on both the MALDI-TOF MS and LC-ESI-MS/MS platforms and also cross-validated between instruments by analyzing the same standard sets and unknowns [28]. As discussed earlier, this cross-validation verified a close agreement between the two different MS platforms.

## 7. Discussion

These activity-based MS analyses have yielded advanced detection methods for diagnosis and detection of anthrax toxins and botulinum neurotoxins with detection limits at 5 pg/mL for LF and as low as 0.05 mouse LD50 for BoNT’s [41], up to 20 times greater analytic sensitivity than the mouse bioassay. The 5 pg/mL detection limit for LF ranges from 2–4,000 times better than detection limits for other recently developed methods for anthrax toxins which range from 0.01–20 ng/mL [39,40,42]. The ultra-low detection limits for the LF MS method has facilitated LF detection and diagnosis in early infection, as early as 12 h after spore exposure in both rabbits and rhesus macaques with anthrax [21,43]. For previously developed methods, anthrax toxins PA and LF, have only been measured in late infection, even with a PA method with fairly low detection limits [42]. The LF method also has excellent precision and accuracy, as described above. The EF method detection limit is almost 1000 times lower than that for LF (manuscript in preparation) and therefore, might detect toxin earlier after exposure than observed for the LF method.

The measurement of LF in rhesus macaques with inhalation anthrax provided the first report of a triphasic kinetics of toxemia. Measurement of LF also tracked the course of clinical treatment in a patient with advanced inhalation anthrax which suggests such measurements might be useful for evaluating the efficacy of anthrax therapeutics [22]. In support of its utility and effectiveness, the LF method has been included as part of the CDC response plan during an anthrax emergency. It is also a required measurement under the investigational new drug protocol for use of the anthrax anti-toxin, anthrax immune globulin intravenous (AIGIV).

The use of these methods for the BoNTs provides several distinct advantages over the mouse bioassay for diagnosis, toxin typing, and subtyping. For example, toxin detection can be multiplexed for detecting toxin types /A, /B, /C, /D, /E, and /F [3]. Multiplexing for the primary BoNT types that affect humans, /A, /B, /E, and /F only requires a maximum volume of 500 µL of toxin-containing serum or food sample [41]. The BoNT in a sample, /A, /B, /E, and/or /F is purified with a single high-affinity antibody with specificity for a region that is homologous in all types /A, /B, /E and /F [41]. The captured BoNT is then reacted with four peptide substrates based on SNAP-25 and VAMP-2 that differentiate these four BoNT serotypes based on differential cleavage of any substrate [41]. For example, certain strains of *C. botulinum* produce more than one BoNT serotype (e.g., Ab, Ba, Af producing strains), which would cleave each of two peptide substrates in specific locations. This dual activity can be easily determined by the described methods in one reaction [41]. However, the mouse bioassay requires several milliliters of sample since it must use 500 µL–1 mL for each individual mouse which will receive either no anti-toxin or individual anti-toxins to A, B, E, and F to determine the type that protects the mouse from botulism. This does not take into account quantification of the BoNT levels. Additional mice and sample volumes are required for mouse LD_50_ quantification. Additional advantages to the MS methods include the ability to obtain detailed toxin type information. After antibody capture and the BoNT substrate cleavage reaction, the toxin captured on the beads can be digested with trypsin and sequenced for delivering information about the specific subtype and even identification of novel subtypes [5,41,44]. The BoNT methods also provide enhanced sensitivity over the mouse for all toxin types. This provides an excellent resource for rapid BoNT detection and toxin-typing during an emergency related to intentional or accidental *C. botulinum* contamination of food or other consumables [45].

We are developing similar toxin activity based methods for *Clostridium difficile* and *Bordetella pertussis*, both of which have gained or regained prominence in recent years as important pathogens causing public health problems. *C. difficile* has developed enhanced virulence and currently only two anti-infectives, metronidazole and vancomycin, are routinely used to treat it [46]. It has been suggested that treatment with fluoroquinolones for other infections may predispose patients to *C. difficile* infection by eliminating the protective intestinal flora [47]. For *B. pertussis*, the re-emergence of infections reported since 1990 in areas with widespread vaccination [48] is presumed to be due to decreased effectiveness of the vaccine [49]. The cause has not been definitively identified but it may be due to shifts in genes for fimbriae and/or waning immunity in adolescents and adults [50]. Methods quantifying the activity of these toxins may be used to evaluate toxin vaccine responses and yield insights into increased virulence.

## 8. Conclusions

The advanced capabilities, speed, and specificity of methods employing both the enzymatic properties of toxins combined with mass spectrometric detection have advanced what we now understand about anthrax infection [6] and botulinum neurotoxins [51]. Application of these methods to toxin quantification for other infections and intoxications may yield novel insights into these emerging public health concerns. Methods which can quantify toxins associated with these pathogens, as well as those causing anthrax and botulism, may provide enhanced diagnostic tests, help understand the nature of changes leading to enhanced virulence, pathogenicity, losses in vaccine efficacy, and provide an accurate measure for evaluating the efficacy of novel therapeutics and vaccines.
